# Comparing mentored research experiences for undergraduates across institutional contexts

**DOI:** 10.1017/cts.2025.59

**Published:** 2025-04-16

**Authors:** Jazzmine K. Waugh, MacKenzie J. Gray, Sanya Surya, Thomas E. Keller

**Affiliations:** 1 Portland State University, Portland, OR, USA; 2 Oregon Health & Science University, Portland, OR, USA

**Keywords:** Mentoring, CTSA, diversity, biomedical workforce, science identity, undergraduate research experiences

## Abstract

**Background:**

Mentored undergraduate research experiences (UREs) can play a critical role in developing science identity and skills, especially for students from historically underrepresented backgrounds. This study investigates science identity and responsibility for scientific roles among scholars in a program aiming to diversify the biomedical workforce. Scholars were placed in UREs at either their home institution (a minority-serving institution [MSI]) or at a research-intensive medical institution with a Clinical and Translational Science Award (CTSA).

**Methods:**

We analyze data from surveys administered annually to the scholars. We first compare changes in science identity for scholars placed at the MSI and the CTSA site from the term after the scholar started their URE to one year later. We then analyze differences in responsibility in scientific roles performed by scholars at the two institutions.

**Results:**

We found evidence of gains in science identity after a year for scholars placed at both institutions but of a somewhat larger magnitude at the CTSA site. However, no significant differences were observed across institutions on science identity at the endpoint. An exploration of scientific roles suggests that scholars at the CTSA site assumed more responsibility in roles related to data curation and analysis, while scholars at the MSI had higher responsibility for resource acquisition-related roles.

**Conclusion:**

These results suggest that CTSA site URE placements may offer distinct opportunities for both identity formation and skill development beyond placements at home institutions. Overall, these results suggest opportunities for partnerships between MSIs and CTSA sites in the training of biomedical researchers.

## Introduction

According to the National Institutes of Health (NIH), communities underrepresented in the U.S. biomedical workforce include individuals who have disabilities, come from socioeconomically disadvantaged backgrounds, or identify as Black, American Indian, Native Hawaiian, Pacific Islander, Alaska Native, or of Hispanic ethnicity [1]. Providing research training opportunities for undergraduates historically excluded from biomedical careers is an important strategy for diversifying the biomedical research workforce [1,2]. A central feature of many training programs is a mentored undergraduate research experience (URE) enabling students to gain competence and confidence as members of research teams [3–5]. Documented benefits of UREs include enhanced research skills, greater self-confidence and sense of belonging in science, higher student retention, and greater likelihood of entering graduate or professional education programs [6–9]. Furthermore, evidence suggests UREs can be particularly beneficial for students historically underrepresented in science and medicine [3,10]. Mentors play a crucial role in URE outcomes by providing essential structure and guidance [11,12]. Mentors orient students to their projects, set expectations, instruct on procedure, offer feedback and constructive assistance, and support students in developing the traits and perspectives of a scientific researcher [13,14]. For students, the nature and quality of mentoring received in the URE influences their research engagement, gains in research knowledge and skills, and development of self-efficacy and science identity [15–17]. For example, mentees who report receiving more instrumental (task-based) and socioemotional mentoring have been found to have higher science identity [18]. Furthermore, longer-lasting mentored UREs may be more beneficial for students, as they can acquire greater understanding of the research process and develop stronger attributes as a researcher [17,19].

Faculty choose to mentor undergraduate researchers based not only on personal values but also multiple institutional factors: levels of encouragement and recognition from leadership; financial incentives; time and workload constraints; and consideration in promotion reviews [20,21]. In one study, faculty engagement in undergraduate research mentoring was most strongly associated with perceived institutional support for this activity [22]. Furthermore, the emphasis of faculty on research and scholarly productivity more generally is influenced by a variety of institutional characteristics (e.g., expectations, culture, infrastructure, rewards, and opportunity structures) that contribute to a conducive environment for research [23,24].

It stands to reason that the nature, quality, and outcomes of mentored undergraduate research experiences may vary according to the institutional contexts in which they are situated. Although there have been few cross-institutional investigations of this question, one study found student participation in undergraduate research was associated with institution size, selectivity, Carnegie Classification, and aggregated indicators of faculty time spent on research and faculty importance placed on research [25]. Other research indicated that racially/ethnically underrepresented students are less likely to be retained in biomedical and behavioral science degrees at more selective, “higher status” universities, with historically black colleges and universities being an exception [26]. Likewise, another study reported undergraduates at research-intensive institutions were more likely to leave their UREs prematurely than students at primarily masters or undergraduate institutions [27]. Additional research suggests structural differences between institutions shape the relationships undergraduates in biomedical and behavioral sciences have with faculty mentors, with less frequent and less personal interactions at more selective universities and universities with lower undergraduate enrollment [28].

One goal of the Clinical and Translational Science Awards (CTSA) Program, which supports efforts at leading research-intensive medical institutions across the nation, is to advance science through education, training, and career development at all levels [29]. CTSA sites host numerous career development awards for early-career scientists, offer a range of research education and training programs, and emphasize high-quality research mentoring to support the next generation of scientists [30,31]. Given their established programs and infrastructure, CTSA sites would seem well-positioned to support the research training of underrepresented undergraduates. Partnerships between CTSA institutions and minority-serving institutions (MSIs) have been established to provide enhanced training opportunities for historically underrepresented scholars [32]. Indeed, many training programs situated in universities with less research activity are structured to provide URE placements in R1 partner institutions for students to gain exposure to these high research activity settings (e.g., the NIH T34 Undergraduate Research Training Initiative for Student Enhancement or “U-RISE” program).

The current study investigates how the mentored research experiences of historically underrepresented undergraduates may vary by institutional context. Previous cross-institutional studies have not disentangled the influence of institutional context from the programs providing URE opportunities for students because program models, practices, and implementation likely vary by institution. In addition, other potentially confounding factors, such as participating student populations, are rarely controlled. In the current study, students participating in the same research training program are compared according to the institution at which they complete their UREs, either their home institution (an MSI) or a CTSA site. This research design, though not based on random assignment, comes closer to identifying the effects of institutional context because the students are from the same institution and have the same program support, preparation, and expectations for their research experiences. Furthermore, the mentors across both institutions made the same program commitments and received the same mentor training curriculum.

We first examine whether institutional context is associated with change over time in students’ science identity during their research placement. Research has demonstrated that interactions with the environment, and the science context constructed within that environment, has implications for science identity development [33]. Next, we explore potential differences across institutions in the roles and responsibilities mentors assign to undergraduates working on their research projects. Relatively limited research explores how students actually engage in research activities and the types of knowledge and skills they gain [e.g., 7, 11, 34, 35]. The institutional context may impact opportunities available for students to develop skills and make contributions to their research teams [27]. Understanding whether, and how, research experiences vary by institution has implications for improving undergraduate research training programs by prioritizing research in more beneficial settings or providing more tailored support for students for their particular research setting.

## Materials and methods

### Intervention

#### Undergraduate research training program

The Enhancing Cross-disciplinary Infrastructure and Training at Oregon (EXITO) project aims to increase the recruitment and retention of undergraduates from diverse backgrounds in biomedical research [36]. EXITO was funded by the NIH as part of the Building Infrastructure Leading to Diversity (BUILD) initiative [2]. Portland State University (PSU) leads this comprehensive, three-year program for students aspiring to health-related research careers, in partnership with ten other academic institutions. By integrating multiple program components, such as a structured curriculum, mentoring and advising, UREs, and a trainee financial support package, the initiative seeks to help scholars graduate well prepared for graduate school or transition into the biomedical workforce [36].

A core component of EXITO is placement in a Research Learning Community (RLC) for a long-term, intensive research experience. RLCs are faculty-led research teams that have funded research and permit scholars to participate in authentic research, making meaningful contributions to project objectives in actual laboratory settings (in contrast to course-based research). Each RLC has a principal investigator and typically includes additional researchers and project staff, such as other faculty, postdocs, graduate students, and/or undergraduates. Scholars interview prospective RLCs and are matched based on their ranked preferences. They receive support as they transition into RLC placements the summer before their junior year. During the academic year, they work 10 hours per week, followed by an intensive summer research experience of 30 hours per week. In their senior year, they resume working 10 hours per week. RLCs represent a broad range of research, including clinical and translational, biomedical, behavioral, social, and bioengineering research.

#### Institutional contexts

EXITO scholars attending PSU can be placed in RLCs at one of two institutions: their home institution, PSU, or a CTSA site, Oregon Health & Science University (OHSU). Both PSU and OHSU are public postsecondary institutions located in Portland, Oregon with a Carnegie Classification of “R2: High Research Spending and Doctorate Production.” However, OHSU has a much higher volume of research activity and external funding. While the two institutions report finances differently, PSU reported “research expenditures” of $95.5 million in 2023 [37], whereas OHSU reported “research funding” of $595.9 million [38]. OHSU has a much lower proportion of undergraduates; as of Fall 2023, OHSU had 788 undergraduate students (∼28% of all students) and 2,064 professional or graduate students (∼72%) [39], while PSU had 16,423 undergraduate students (∼78% of all students) and 4,617 graduate students (∼22%) [37]. OHSU students are predominantly white (∼61%) [39], while PSU is designated as an MSI with just under half of students identifying as white [37]. Differences between these institutions may contribute to variation in EXITO scholar research experiences.

### Study design

The study uses self-reported survey data from scholars for secondary analyses comparing their experiences of participating in RLCs at PSU or OHSU. The first comparison focuses on longitudinal change in level of science identity between two timepoints—initially after 1–2 months in the RLC placement and then again one year later. The second, cross-sectional comparison focuses on the nature of work performed in RLCs as reported near the end of the placement experience.

### Sample

Between the first cohort in 2015 and the present, there have been eight cohorts and 628 scholars in EXITO. However, we limited analyses to scholars who were enrolled in the program as PSU students and had placements at PSU or OHSU (n = 479). Next, we limited analyses to data collected from scholars in Spring 2020 or before (i.e., the first four cohorts, *n* = 312), as after this time institutions went remote due to the COVID-19 pandemic, potentially dramatically altering research participation. In addition, scholars are permitted to change RLC placements once. Therefore, to avoid confounding results, scholars who changed placements from OHSU to PSU or vice versa were also excluded. Sample sizes were further limited by data constraints associated with each analysis. Analysis of change in science identity was restricted to scholars reporting at both timepoints (n = 84). Comparison of research roles and activities was restricted to scholars reporting in their senior year (after approximately 1.5 years of being in the placement) so that the full range of their activities could be captured (*n* = 123).

Demographic characteristics of the respective samples are reported by RLC institutions in Table 1 based on self-reported information provided on the application to EXITO. Due to the goals and recruitment process of EXITO, this sample reflects individuals underrepresented in the biomedical workforce [36], including students who identify as racial and ethnic minorities, have disabilities, or experience social or economic disadvantages. The demographic characteristics of the analytic sample did not differ when compared to all scholars with an RLC at PSU or OHSU during the same time period (i.e., first four cohorts of EXITO) (SM Table 1). We also found no evidence of differences in the demographics of participants at OHSU versus PSU within either analytic sample (SM Table 2).

**Table 1.** Self-reported demographics for participants who responded to the YASS and CREDIT URE surveys and were in PSU or OHSU research placements. Percentages are calculated out of the number of scholars in the sample for each survey at each institution. Percentages may not sum to 100 due to rounding

	YASS		CREDIT URE	
Category	OHSU (%) (n = 36)	PSU (%) (n = 48)	OHSU (%) (n = 60)	PSU (%) (n = 63)
**Gender**				
Female	26 (72.2%)	28 (58.3%)	43 (71.7%)	43 (68.3%)
Male	9 (25.0%)	19 (39.6%)	16 (26.7%)	18 (28.6%)
Non-binary/ third gender	1 (2.8%)	1 (2.1%)	1 (1.7%)	2 (3.2%)
**Race/Ethnicity**				
Asian	9 (25.0%)	6 (12.5%)	12 (20.0%)	6 (9.5%)
Underrepresented race/ethnicity	10 (27.8%)	15 (31.3%)	16 (26.7%)	21 (33.3%)
Unknown	2 (5.6%)	2 (4.2%)	4 (6.7%)	2 (3.2%)
White	15 (41.7%)	25 (52.1%)	28 (46.7%)	34 (54.0%)
**First-generation status**				
First generation	23 (63.9%)	34 (70.8%)	38 (63.3%)	43 (68.3%)
Continuing generation	13 (36.1%)	14 (29.2%)	22 (36.7%)	20 (31.8%)

“Unknown” indicates scholar left the field blank or declined to respond. “First generation” indicates parents/guardians did not receive a Bachelor’s degree. “Continuing generation” indicates a parent/guardian received a Bachelor’s degree. “Underrepresented” indicates scholar from a race/ethnicity underrepresented in biomedical sciences as identified by NIH [1]: Black/ African American, Hispanic/ Latino, American Indian/ Alaska Native, and Native Hawaiian and other Pacific Islander.

### Procedures

Scholars recruited for EXITO completed a competitive application process, and selected participants were enrolled in the program. At the program orientation, scholars were invited to voluntarily participate in the evaluation study for the intervention, and informed consent was obtained according to Institutional Review Board-approved procedures. As part of EXITO evaluation, scholars were asked to complete a Yearly Academic Scholar Survey (YASS) in the fall of each academic year. For students in RLCs, this survey administration would occur 1–2 months after placement (T1: beginning of junior year) or 13–14 months after placement (T2: beginning of senior year). Another survey focusing on placement experiences, including research roles, was piloted in 2016 and administered each spring through 2021. Consequently, this annual survey assessed scholars near the end of the junior and/or senior year. Surveys were distributed to EXITO scholars through email links to surveys, and paper surveys were administered during in-person events. No incentives were offered for completion of these surveys but responses were encouraged as an expectation of EXITO participation.

### Measures

#### Science identity

Scholars’ science identity was measured on the YASS with a modified version of the Science Identity Scale from the Tripartite Integration Model of Social Influence [40]. Scholars were asked to rate the extent to which four science identity-related statements felt true to them on a scale of 1 (strongly disagree) to 5 (strongly agree). The statements were “I have a strong sense of belonging to a community of scientists,” “I derive great personal satisfaction from working on a team that is doing important research,” “I think of myself as a scientist,” and “I feel like I belong in the field of science.” We found support for the use of all four statements with Confirmatory Factor Analysis (SM Table 3). Assessing reliability with McDonald’s Omega [41] indicated the scale was acceptably reliable, with an Omega total value of 0.85. The science identity score was derived by summing the four items.

#### Scientific roles

*S*cholars’ contributions to research projects were assessed with the Contributor Roles Taxonomy Undergraduate Research Experience (CREDIT URE) [42]. The CREDIT URE defines 14 non-exclusive roles that undergraduate researchers might engage in (SM Table 4). The 14 roles are: conceptualization (formulation of research goals); data curation (annotating, cleaning, and maintaining data); formal analysis (statistical analysis and data synthesis); funding acquisition (grant-writing); investigation (performing experiments and/or collecting data); methodology (development of methods); project administration (management of research activity); resources (acquisition of study materials and tools); software (testing and designing code); supervision (oversight of research activity, including mentorship external to the core team); validation (verification of research results and reproducibility); visualization (data visualization); writing – original draft, and writing - reviewing and editing. For each role, the scholar and/or their faculty mentor rates the scholars’ participation on a scale of: 0 = no responsibility; 1 = little responsibility; 2 = moderate responsibility; 3 = primary responsibility, with an additional option for “I don’t know.” Because mentor and mentee responses have been found to have a high degree of concordance for EXITO scholars on this measure [42], we chose to focus on mentee responses to center on the scholar experience.

### Analysis

To investigate whether change in science identity during RLC placement varied by institutional context, we analyzed data from students who had responded to the YASS survey at two timepoints: the term after they entered their RLC (T1), and one year after the first survey (T2). To evaluate meaningful change over time, we used paired-samples t-tests comparing T1 and T2 science identity scores for the scholars at PSU and repeated this procedure for the scholars at OHSU. To evaluate differences between institutions, we used independent-samples t-tests comparing PSU and OHSU scholars on science identity scores at both T1 and T2. We also performed a two-way ANOVA to investigate the effects associated with time, institution, and the interaction between the two on science identity.

To better understand potential differences in the nature of research roles and activities in RLC placements, we conducted t-tests comparing scholars at PSU and OHSU on each of the CREDIT URE items assessed in the senior year (in RLCs for ∼ 1.5 years). We did not correct for performing multiple tests as this analysis was exploratory, not confirmatory [43]. If scholars responded “I don’t know” for a role, they were not included in calculations for that role but were included in calculations for other roles. Out of the scholars included in this analysis, for each role, “I don’t know” was selected between 0 times (for software, 0% of the 123 responses) and 7 times (for visualization, 5.7% of the 123 responses).

If scholars were more likely to change RLC placements, necessitating adaptation to a new research environment, or more likely to persist in the program at one institution than the other, it could be responsible for any differences observed between institutions. To explore this, we used a chi-square test to compare the number of students who changed RLC placements at PSU versus OHSU. A second chi-square test compared the number of scholars who completed EXITO in PSU and OHSU RLCs.

All analyses were conducted in R (version 4.4.2 [44]). We calculated MacDonald’s Omega using the package “psych” [45]. We conducted chi-square, ANOVA, and t-tests with the “stats” package in base R. Significance for all tests was assessed with α = 0.05.

## Results

**Figure 1.**
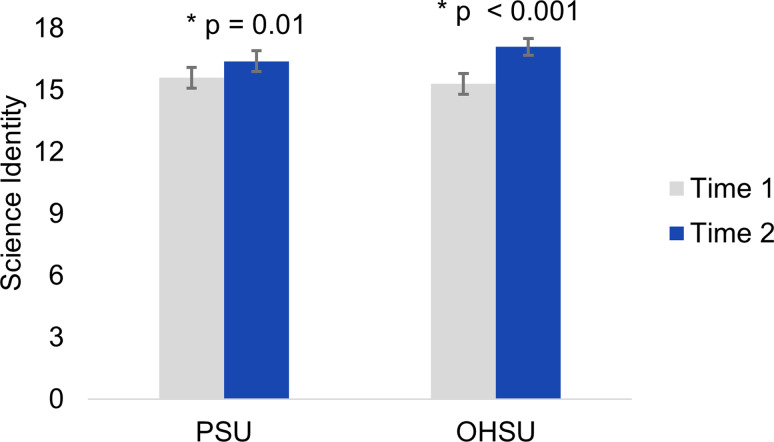
Change in science identity over time by institution. Science identity is considered at two time points: the term after a scholar was placed in their research learning community (“RLC”) (“Time 1”) and approximately one year later (“Time 2”), for both PSU (n = 48) and OHSU (n = 36). Error bars show standard error of the mean. Significance was tested within each institution, time 2 versus time 1. * denotes significance at α=0.05.

A total of 84 scholars reported their science identity at two time-steps: the term after they joined their RLC and approximately one year after the first survey. For scholars in OHSU RLCs, the increase in science identity was statistically significant (T1: Mean (M) = 15.3, standard error of the mean (SE) = 0.5; T2: *M* = 17.1, SE = 0.4) (Fig. 1). The increase in science identity for scholars at PSU RLCs also was statistically significant (T1: *M* = 15.6, SE = 0.5; T2: *M* = 16.4, SE = 0.5) but not of the same magnitude observed at OHSU. We found no evidence of a difference between PSU and OHSU scholars in starting (T1) values (t [80] = 0.65, *p* = 0.52) nor in ending (T2) values (t [80] = 0.96, *p* = 0.34). A two-way ANOVA confirmed a statistically significant difference in science identity associated with change over time (F [1] = 6.9, *p* = 0.009), but not institution (F [1] = 0.02, *p* = 0.88), nor the interaction between time and institution (F [1] = 1.2, *p* = 0.28).

**Figure 2.**
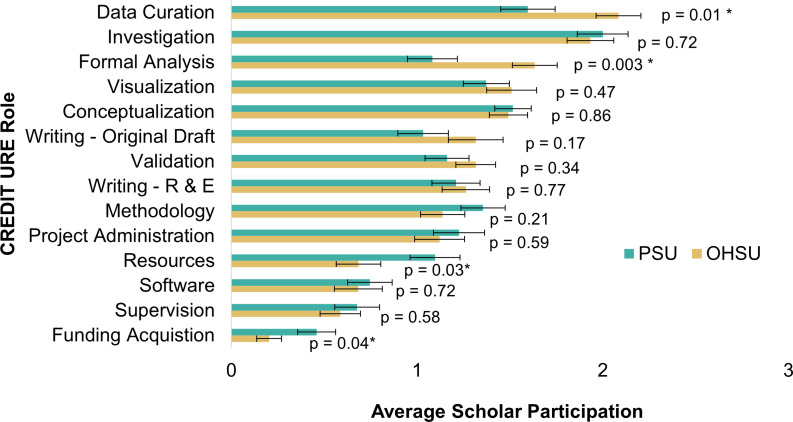
Scholar responsibility in research roles. Average scholar participation in CREDIT URE roles as reported by scholars at PSU (n = 63) and OHSU (n = 60) after being in their RLC placements for ∼ 1.5 years. For each role, scholars rate their own participation on a scale of: 0=no responsibility; 1=little responsibility; 2=moderate responsibility; 3=primary responsibility, with an additional option for “I don’t know.” Data sorted from highest to lowest average participation at OHSU. P-values are labeled for each role; * denotes significantly different participation reported by scholars at OHSU compared to PSU at α=0.05. “Writing R & E” = writing-reviewing and editing. Error bars show standard error of the mean.

CREDIT URE survey responses were received from 63 scholars placed at PSU and 60 placed at OHSU during the spring term of their senior year. Considerable variation was observed in the mean level of responsibility for the different CREDIT URE roles, but all roles were represented in the reports of at least some scholars at both PSU and OHSU (Fig. 2). Across both schools, scholars tended to report highest responsibility in roles involving data collection and annotation (i.e., “data curation” and “investigation”), formal analysis, and visualization. They tended to participate less in roles involving oversight (i.e., “supervision” and “project administration”) and resource provision (i.e., “funding acquisition” and “resources”).

In our exploratory analysis, we found evidence of differences between institutions in four roles: data curation, formal analysis, resources, and funding acquisition. Scholars at OHSU reported higher responsibility in roles related to data curation (OHSU: *M* = 2.1, SE = 0.1; PSU: *M* = 1.6, SD = 0.2) and formal analysis (OHSU: *M* = 1.6, SE = 0.1; PSU: *M* = 1.2, SD = 0.2). On the other hand, scholars at PSU reported higher responsibility in roles relating to resources (OHSU: *M* = 0.68, SE = 0.12; PSU: *M* = 1.10, SE = 0.13) and funding acquisition (OHSU: *M* = 0.20, SE = 0.07; PSU: *M* = 0.46, SE = 0.10).

We found no evidence of differences in rates of scholars changing RLC placements within institutions: 15/68 of PSU participants changed labs, while 10/69 scholars at OHSU changed labs (χ^2^ = 0.86, *p* = 0.35). Similarly, we found no evidence of differences in retention rates between scholars at the two institutions: 61/68 scholars at PSU completed EXITO versus 57/69 scholars at OHSU (χ^2^ = 0.91, *p* = 0.34). As a result, differences in persistence do not appear to be responsible for differences in science identity and research-related responsibilities between the two institutions.

## Discussion

This study explored the identity development and research-related responsibilities of a diverse group of undergraduate scholars in mentored research placements at two types of institutions. One institution is a CTSA site with a focus on medical research, while the other is a public research MSI and the home institution of the scholars. Gains in science identity after a year of mentored research were statistically significant for scholars placed at both institutions. Greater increases in science identity were observed for scholars at the CTSA site, but the interaction between institution and time was not statistically significant. Furthermore, the difference in science identity between institutions at the endpoint (T2) was not statistically significant. The scholars who participated in this study reported involvement in all fourteen CREDIT URE roles at both institutions, with high levels of agreement between scholars at both institutions on their level of responsibility for the majority of these roles. However, scholars at the CTSA institution reported more responsibility in roles related to data analysis (data curation and formal analysis), while scholars at the home institution reported more responsibility in roles related to resource acquisition (including resources and funding acquisition). Overall, in contrast to previous research suggesting UREs may be compromised at high status research-intensive institutions [26–28], we found evidence that engaging in a mentored research experience at a CTSA institution may provide distinct opportunities for science identity development and participation in a wide variety of scientific roles.

Our detection of improvement in science identity over the course of a mentored URE is consistent with previous findings [46]. However, the current study is noteworthy for indicating differences in the growth of science identity between institutional contexts for students participating within the same program. Although the beginning and ending means in science identity did not differ significantly by institution, the CTSA values were initially slightly lower and then subsequently higher than those at the home institution. The first assessment of science identity was shortly after scholars had been placed with their RLCs, and therefore may reflect an effect of their early experiences in their research placement. In other words, initial levels of science identity may have been marginally suppressed by scholars at the CTSA site adapting to the transition to an unfamiliar environment and potentially feeling intimidated in a research-intensive context [26,27,47]. It appears scholars who persevered in their RLCs at the CTSA site showed strong gains in science identity, as would be expected with continuing in longer-lasting mentoring experiences that provide opportunities to engage more deeply in research endeavors [19,34].

Conceptually, science identity is developed by acquiring scientific knowledge (competence), executing scientific practices (performance), and seeing oneself and/or being acknowledged by others as a science person (recognition) [48]. *In situ* experiences with active research teams may contribute to science identity through these avenues depending on the actual roles and responsibilities afforded scholars by their mentors [49]. The CREDIT URE highlights the multiple opportunities for practical learning and skill development for students in these real-world research settings. While not explicitly examined in this study, prior research on this population demonstrated high levels of agreement between scholars and their research mentors regarding responsibility in these research-related roles [42], suggesting mentors may be supporting science identity development by providing recognition. Our exploration revealed that scholars placed in both institutions gained experience and made contributions across a broad range of activities, with some level of responsibility reported across all fourteen roles captured by the CREDIT URE.

However, we discovered some distinctions based on institutional context. Scholars at the CTSA institution had higher responsibility for data curation and formal analysis, whereas scholars engaged in undergraduate research at their home institution had higher responsibility for resources and funding acquisition. These differences likely reflect the CTSA site having greater infrastructure for securing and managing external grants and consequently having more ongoing projects, allowing scholars to focus on working with data. For novice researchers, working with data may have fostered a stronger sense of “doing science.” [49] In contrast, scholars at the home institution may have provided more administrative support to mentors attempting to initiate projects, which provides an understanding of the realities of securing resources to conduct research.

The findings of this study may diverge from previous research on cross-institutional factors affecting UREs because all scholars were participating in the same research training program, enabling us to better discern the role of institutional context. Although UREs at research-intensive institutions generally have been reported as less hospitable for historically underrepresented students [26–28], EXITO’s scholars may have had more positive research experiences at the CTSA site because the program featured components designed to support scholars before and after entering this research setting [36]. For example, prior to entry into an RLC, scholars participated in a year of preparation that included coursework on the fundamentals of research and regular workshops on professional development, skills development, and orientations to lab expectations [36]. Likewise, a multiple mentor model provided scholars with support and guidance from faculty and peer mentors in addition to their research mentors [50]. This holistic approach was intended to help scholars build confidence for working in the CTSA research environment and to offer ongoing resources to maintain the longevity of their placements. Similarly, although mentors at OHSU may have been likely less to have experience working with undergraduates, the program provided structure and support that included an orientation and documentation outlining guidelines and expectations, an evidence-based mentor training curriculum, and regular check-in logs to identify and address any concerns. Such program practices and infrastructure may be particularly important when students are navigating research experiences across different institutional contexts.

### Limitations

The findings reported here should be interpreted cautiously given several limitations of our study. First, the experiences of scholars in the study may not be reflective of all undergraduate researchers. EXITO scholars must be accepted into the program to participate; many of these scholars are already high achieving. In addition, not all scholars chose to complete the surveys, and there may be important differences between those who did and did not agree to participate, although we found no evidence of demographic differences.

Second, scholars in RLC placements at the two institutions may also differ, as scholars indicate their preferences for placements and are not randomly assigned to institutions. However, scholars had similar initial levels of science identity across institutions, and we did not find evidence of demographic differences among scholars at the two institutions.

Third, our analysis did not include other potentially relevant variables pertaining to scholars or their placements. We do not know whether previous research experience on the part of the scholars, or mentoring experience on the part of the mentors, may differ systematically between the two institutions. However, we note that scholars at both institutions and their mentors were given access to identical support as noted above. We also omitted characteristics of RLCs that may affect scholar experiences or outcomes.

Finally, our measure of science identity may not capture all of the ways in which a scholar can identify as a scientist. For instance, scores may have been higher at one or both institutions if scholars were asked about their identity as a “clinical researcher” or another more clinically focused term. Overall, our results should not be interpreted as necessarily indicative of all undergraduate researchers or all the ways that an undergraduate researcher can engage with science.

## Conclusion

The findings of this study suggest the value of future research investigating the significance of institutional context on the mentored research experiences of undergraduates. Comparing students within the same research training program, we found evidence of gains in science identity in undergraduates mentored at both the CTSA and MSI institutions, with the degree of increase somewhat favoring the CTSA institution. We also identified some differences in responsibility for scientific roles between scholars at the CTSA institution versus their home institution, an MSI. More research is needed to learn how the roles scholars perform when working with their research mentors might contribute to their development as scientists. Furthermore, the findings indicate a need for further exploration of how research experiences within research-intensive medical institutions may offer distinct advantages from other types of institutions in the training of undergraduates. Overall, this study contributes preliminary support for continued placement of scholars at research-intensive institutions for UREs, although it may be important to ensure scholars entering these settings have proper program support.

## Supporting information

Waugh et al. supplementary material Waugh et al. supplementary material

## Supplementary material

The supplementary material for this article can be found at https://doi.org/10.1017/cts.2025.59.
