# Supplementary material for: Comparing mentored research experiences for undergraduates across institutional contexts
Source: J Clin Transl Sci. 2025 Apr 16;9(1):e95. doi: 10.1017/cts.2025.59 (PMC12089855; doi:10.1017/cts.2025.59)
Supplement: Waugh et al. supplementary material [file S2059866125000597sup001.docx]

Supplementary Material for Waugh et al. 2025

# Demographics Comparison

We explored whether our survey participants were representative of EXITO scholars as a whole by comparing the demographics of those who participated in at least one of the surveys (YASS or CREDIT URE) to demographics of EXITO scholars within the same time period (i.e., the first four cohorts of EXITO) who participated in a Research Learning Community at Portland State University (PSU) or Oregon Health & Science University (OHSU) (SM Table 1). We used Fisher’s exact test due to the small sample size [1]. There was no evidence of significant differences between the three groups (i.e., EXITO, PSU, and OHSU) for race/ethnicity, generation status, or gender. All Fisher’s exact tests were conducted using the stats package in base R.

| SM Table 1: Self-reported demographics for those who responded to the YASS and/or CREDIT URE surveys and that were placed in Portland State University (PSU) or Oregon Health & Science University (OHSU) Research Learning Communities (RLCs), as well as for EXITO (constrained to EXITO scholars within the same time period [i.e., the first four cohorts of EXITO] who participated in an RLC at PSU or OHSU). For survey demographics, percentages are calculated out of the number of scholars in the sample for each survey at each institution. Percentages may not sum to 100 due to rounding. All EXITO-wide demographics considered were not significantly different from survey participant demographics (generation status: *p* = 0.93; race/ethnicity: *p* = 0.31; gender: *p* = 0.94). | | | |
| --- | --- | --- | --- |
| Category | EXITO Ct (%)  (n = 259) | PSU Ct (%)  (n = 68) | OHSU Ct (%)  (n = 69) |
| **Gender** | | | |
| Female | 174 (67.2%) | 44 (64.7%) | 48 (69.6%) |
| Male | 80 (30.1%) | 22 (32.4%) | 20 (29.0%) |
| Non-Binary/ Third Gender | 5 (1.9%) | 2 (2.9%) | 1 (1.5%) |
| **Race/Ethnicity** | | | |
| Asian | 36 (13.9%) | 7 (10.3%) | 15 (21.7%) |
| Underrepresented R/E | 121 (46.7%) | 34 (50.0%) | 26 (37.7%) |
| Unknown | 21 (8.1%) | 2 (2.9%) | 4 (5.8%) |
| White | 81 (31.3%) | 25 (36.8%) | 24 (34.8%) |
| **Generation Status** | | | |
| First generation | 168 (64.9%) | 46 (67.7%) | 46 (66.7%) |
| Not first generation | 91 (35.1%) | 22 (32.4%) | 23 (33.3%) |
| “Unknown” indicates scholar left the field blank or declined to respond. “First generation” indicates parents/guardians did not receive a Bachelor's degree from college. “Underrepresented R/E” indicates scholar from a race/ethnicity underrepresented in biomedical sciences as identified by National Institutes of Health: Blacks/ African Americans, Hispanics/ Latinos, American Indians/ Alaska Natives, and Native Hawaiians and other Pacific Islanders. | | | |

We then explored whether there were significant differences between PSU and OHSU scholars within each survey (SM Table 2). We used Fisher’s exact test again, due to small sample sizes. We found no evidence of significant differences within either survey for gender, race/ethnicity, or generation status.

| SM Table 2: P-value results for Fisher’s exact tests for gender, race/ethnicity, and generation status. The tests compare demographics of participants from OHSU versus PSU, within one survey (either CREDIT URE or YASS). | | |
| --- | --- | --- |
|  | **Institutions** | |
| **Demographic** | CREDIT URE | YASS |
| Gender | 0.41 | 0.94 |
| Race/Ethnicity | 0.51 | 0.27 |
| Generation Status | 0.64 | 0.58 |
| “Generation status” indicates whether the student is first generation or not (i.e., whether parents/guardians of the student received a Bachelor's degree from college). | | |

# Yearly Academic Scholar Survey (YASS) Confirmatory Factor Analysis

We performed Confirmatory Factor Analysis [2] with the package Lavaan [3] for the four science identity items on the YASS to examine the factors influencing scholar science identity. The four items were: “I have a strong sense of belonging to a community of scientists,” “I derive great personal satisfaction from working on a team that is doing important research,” “I think of myself as a scientist,” and “I feel like I belong in the field of science.” As all of the items were derived from the Science Identity Scale from the Tripartite Integration Model of Social Influence, we hypothesized the existence of one factor. With all four items included, the model fit appeared to be acceptable (SM Table 3).

| SM Table 3: Results of Confirmatory Factor Analysis with all four items: “I have a strong sense of belonging to a community of scientists,” “I derive great personal satisfaction from working on a team that is doing important research,” “I think of myself as a scientist,” and “I feel like I belong in the field of science.” | |
| --- | --- |
| Root Mean Square Error of Approximation (RMSEA) | 0.07 |
| Comparative Fit Index (CFI): | 1.00 |
| Tucker-Lewis Index | 1.00 |
| Standardized Root Mean Square Residual (SRMR) | 0.03 |

**CREDIT URE Scholar Survey**

Scholars placed in Research Learning Communities were asked to complete the CREDIT URE survey describing their level of responsibility in 14 scientific roles (SM Table 4).

| SM Table 4: All items on the CREDIT URE survey for scholars. For the 14 CREDIT URE roles, scholars were given the instructions “Please complete the survey below regarding your role(s) within your Research Learning Community lab experience. This survey is designed to assess your contribution to your lab using a preset list of roles and responsibility levels. Please choose the best answer for each of the role types listed below: No Responsibility, Little Responsibility, Moderate Responsibility, Primary Responsibility, I Don't Know.” |
| --- |
| 1) Today's date |
| 2) Conceptualization (Ideas; formulation or evolution of overarching research goals and aims) |
| 3) Data Curation (Management activities to annotate (produce metadata), clean data and maintain research data (including software code, where it is necessary for interpreting the data itself) for initial use and later re-use) |
| 4) Formal Analysis (Application of statistical, mathematical, computational, or other formal techniques to analyze or synthesize study data) |
| 5) Funding Acquisition (Acquisition of the financial support for the project leading to this publication) |
| 6) Investigation (Conducting a research and investigation process, specifically performing the experiments, or data/evidence collection) |
| 7) Methodology (Development or design of methodology; creation of models) |
| 8) Project Administration (Management and coordination responsibility for the research activity planning and execution) |
| 9) Resources (Provision of study materials, reagents, materials, patients, laboratory samples, animals, instrumentation, computing resources, or other analysis tools) |
| 10) Software (Programming, software development; designing computer programs; implementation of the computer code and supporting algorithms; testing of existing code components) |
| 11) Supervision (Oversight and leadership responsibility for the research activity planning and execution, including mentorship external to the core team) |
| 12) Validation (Verification, whether as a part of the activity or separate, of the overall replication/reproducibility of results/experiments and other research outputs) |
| 13) Visualization (Preparation, creation and/or presentation of the published work, specifically visualization/data presentation) |
| 14) Writing - original draft (Preparation, creation and/or presentation of the published work, specifically writing the initial draft (including substantive translation) |
| 15) Writing - reviewing and editing (Preparation, creation and/or presentation of the published work by those from the original research group, specifically critical review, commentary or revision including pre- or post-publication stages) |
| 16) This survey covers many aspects of a wide variety of labs. Were there any words, phrases, or activities listed above that you didn't understand or needed more explanation? Please share with us! |

**References**

1. **Kim, HY.** Statistical notes for clinical researchers: Chi-squared test and Fisher's exact test. *Restorative dentistry & endodontics*. 2017; **42***:*152.
2. **Knekta, E, Runyon, C, Eddy, S.** One size doesn’t fit all: Using factor analysis to gather validity evidence when using surveys in your research. *CBE—Life Sciences Education*. 2019; **18**: rm1.
3. **Rosseel, Y.** lavaan: An R package for structural equation modeling. *Journal of statistical software*, 2012; **48**: 1-36.
